# Spectral denoising based on Hilbert–Huang transform combined with F-test

**DOI:** 10.3389/fchem.2022.949461

**Published:** 2022-08-30

**Authors:** Xihui Bian, Mengxuan Ling, Yuanyuan Chu, Peng Liu, Xiaoyao Tan

**Affiliations:** ^1^ Key Laboratory of Separation Membranes and Membrane Processes, School of Chemical Engineering and Technology, Tiangong University, Tianjin, China; ^2^ Key Lab of Process Analysis and Control of Sichuan Universities, Yibin University, Sichuan, China; ^3^ State Key Laboratory of Plateau Ecology and Agriculture, Qinghai University, Xining, China

**Keywords:** denoising, Hilbert–Huang transform, empirical mode decomposition, x-ray diffraction, x-ray photoelectron spectrum, f-test

## Abstract

Due to the influence of uncontrollable factors such as the environment and instruments, noise is unavoidable in a spectral signal, which may affect the spectral resolution and analysis result. In the present work, a novel spectral denoising method is developed based on the Hilbert–Huang transform (HHT) and F-test. In this approach, the original spectral signal is first decomposed by empirical mode decomposition (EMD). A series of intrinsic mode functions (IMFs) and a residual (**r**) are obtained. Then, the Hilbert transform (HT) is performed on each IMF and **r** to calculate their instantaneous frequencies. The mean and standard deviation of instantaneous frequencies are calculated to further illustrate the IMF frequency information. Third, the F-test is used to determine the cut-off point between noise frequency components and non-noise ones. Finally, the denoising signal is reconstructed by adding the IMF components after the cut-off point. Artificially chemical noised signal, X-ray diffraction (XRD) spectrum, and X-ray photoelectron spectrum (XPS) are used to validate the performance of the method in terms of the signal-to-noise ratio (SNR). The results show that the method provides superior denoising capabilities compared with Savitzky–Golay (SG) smoothing.

## Introduction

As a fast, non-destructive analytical technique, spectral analysis plays an increasingly important role in the fields of traditional Chinese medicine (TCM) (Ma et al., 2020), food (Bian et al., 2017; Amendola et al., 2020), bio-medicine (Wu et al., 2021; Yang et al., 2022), and the environment (Sipponen and Osterberg, 2019), etc. However, spectra often contain noise from instruments and operational errors. Instrumental noise mainly includes dark noise caused by the thermal effect, photon noise caused by the photon hitting detector, and electronic noise caused by the A/D converter and circuit board error. In addition, non-standard experimental operations can also produce noise (Mishra et al., 2020). The noise could obscure some useful information in the spectra, which results in low resolution and prediction accuracy. Therefore, it is essential to remove noise from the spectra without unduly reducing the underlying information (Chen et al., 2004).

The commonly used spectral signal denoising methods are Savitzky–Golay (SG) smoothing (Savitzky and Golay, 1964) and wavelet transformation (WT) (Shao et al., 2002). SG smoothing is a filtering method based on local polynomial least square fitting in the time domain, also known as convolution smoothing, which has been developed from the moving average method (Zhang et al., 2019). The SG smoothing is a weighted average method that emphasizes the role of the center point (Chu X. L. et al., 2022). A symmetric window of *i* = 2*ω*+1 is used, where *i* and *ω* are the points of the moving window (i.e., window size) and half window width, respectively. The smoothed value at wavelength *k* is

$${\text{x}}_{k,\text{smooth}}={\bar{\text{x}}}_{k}=\frac{1}{\text{H}}\sum_{i=-\omega }^{+\omega }{\text{x}}_{k+i}h_{i},$$ (1)

where *h*_*i*_ is the smoothing coefficient, which can be obtained by polynomial fitting based on the least square. H is the normalized factor $\text{H}=\sum_{i=-\omega }^{+\omega }h_{i}$ . Most of the noise and interference of abnormal points can be removed by SG smoothing. Moreover, the peak shift of the spectrum is overcome without delays. However, SG smoothing is only performed once for noise removal, which cannot remove noise completely from the spectrum with in-homogeneous frequency. Compared with SG smoothing, WT is more refined and efficient because it decomposes the original spectra into details and approximations with different frequencies step by step (Shao et al., 2019). Many wavelet functions such as Haar, Daubechies, Symlets, and Coiflets have been developed for WT (Fan et al., 2017). In recent decades, WT has become quite a useful tool for signal processing in analytical chemistry (Bian et al., 2011). However, abundant wavelet functions and decomposition scales also make it difficult to select the parameters for WT (Chen et al., 2011).

To overcome the drawbacks of WT, Huang et al. (1998) introduced the Hilbert–Huang transform (HHT), which includes empirical mode decomposition (EMD) and Hilbert transform (HT). EMD can decompose any complex signal into a finite number of intrinsic modal function components (IMFs) and a residual (Bian et al., 2016). Compared with WT, the decomposition of EMD does not require any predefined basis function, which is adaptive and can be applied to any signal (Wang et al., 2018; Yao et al., 2019). Then, HT is performed on each IMF, and their corresponding instantaneous frequencies can be obtained (Peng et al., 2005). HHT denoising has been successfully used in fault diagnosis (Jin et al., 2015; Fan et al., 2022), speech recognition (Krishna and Ramaswamy, 2017), biomedical signal (Lin and Zhu, 2012; Chen et al., 2021), geophysics (Tang et al., 2015; Chen et al., 2017), and so on. However, spectral denoising by HHT is seldom used in analytical chemistry. Moreover, previous studies have determined the cut-off point between noise and useful signal by observing IMF components. However, it is difficult to distinguish. Therefore, it is crucial to determine the cut-off point between high and low frequencies.

In this research, an effective method based on HHT and F-test is proposed to eliminate the noise from the noisy spectral signal. Initially, EMD is introduced to decompose the original spectrum. A series of IMFs from high to low frequencies are obtained. Then, HT is applied to each IMF to obtain instantaneous frequencies. The mean instantaneous frequency combined with the F-test is used to determine the cut-off point between noise components and non-noise ones. Artificially chemical noised signal, X-ray diffraction (XRD) spectrum, and X-ray photoelectron spectrum (XPS) were used to verify the effectiveness and feasibility of the proposed method. The performance of the method is evaluated by the SNR and compared with SG smoothing.

## Theory and algorithm

### Empirical mode decomposition

**FIGURE 1:**
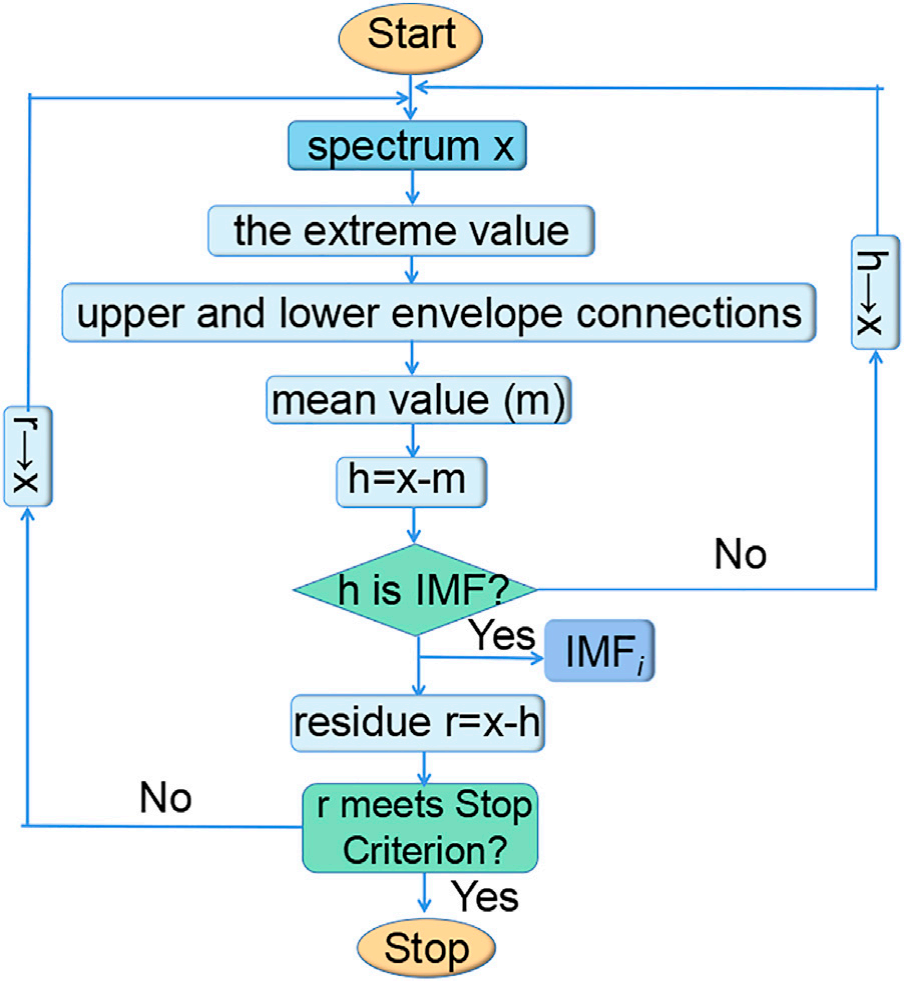
Sifting process of EMD.

EMD is a new adaptive spectral decomposition method. Through a sifting process, EMD can decompose spectra into a certain number of IMFs and a residual. With the increase in IMF orders, the degree of oscillation becomes lower and lower (Bian et al., 2017). The flow chart of the sifting process of EMD is shown in Figure 1.

First, in the sifting process for the spectrum, all local maxima and minima of the original spectrum **x** are connected to form upper and lower envelopes by cubic spline lines, respectively. Then, the mean values **m** of the two envelopes are computed by the simple average. Subsequently, component **h** is computed by which the difference between the original spectrum **x** and mean values **m** can be found. Whether **h** is an IMF by definition is determined. It is worth noting that the IMFs satisfy two conditions. One is that in the whole data set, the number of extreme and zero-crossings must either be the same or different at most by one. The other is that at any point, the mean value of the upper and lower envelopes is zero (Sun et al., 2006). If **h** does not meet the IMF definition, **h**, as a new cycle performs the abovementioned operations until an IMF is obtained. After determining an IMF component, the component **h** is subtracted from the spectrum **x** to get the residual **r**, and whether the residual **r** becomes a monotone function is judged. Finally, the sifting process ends till the residue contains no more than one extreme.

### Instantaneous frequency calculation based on Hilbert transform

Although the oscillations decrease with the increase of IMF orders, it is difficult to know the frequency value of the IMF itself. HT is introduced to calculate the instantaneous frequency of each IMF (Li et al., 2016).

For a signal x(*τ*), its Hilbert transform H(*t*) is defined as

$$\text{H}\left(t\right)=\frac{1}{\text{π}}\text{P}\int_{-\infty }^{+\infty }\frac{\text{x}\left(\tau \right)}{t-\tau }\text{d}\left(\tau \right).$$ (2)

Eq. 2 defines HT as the convolution of x(*τ*) with 1/*t*. Therefore, HT emphasizes the local properties of x(*τ*). Then, P indicates the Cauchy principal value of the singular integral to avoid singularities at t=τ, t=±∞ ; this transform exists for all functions of class L^P^ (Le Van Quyen. et al., 2001).

With this definition, x(*τ*) and H(*t*) form the complex conjugate pair, and an analytic signal, Z(*t*), is obtained,

$$\text{Z}\left(t\right)=\text{x}\left(t\right)+\text{iH}\left(t\right)=\text{a}\left(t\right){\text{e}}^{\text{iθ}\left(t\right)}$$ (3)

in which,

a(t)=[x2(t)+H2(t)]12, (4)

$$\text{θ}\left(t\right)=\text{arctan}\left(\frac{\text{H}\left(t\right)}{\text{x}\left(t\right)}\right),$$ (5)

where a(*t*) is the instantaneous amplitude of x(*τ*), which can reflect the change of energy of x(*τ*) with *t*. θ(*t*) is the instantaneous phase of x(*τ*), and the instantaneous frequency ω(*t*) of signal x(*τ*) can be obtained as follows,

$$\text{ω}\left(t\right)=\frac{\text{dθ}\left(t\right)}{\text{d}\left(t\right)}.$$ (6)

### The proposed denoising method

**FIGURE 2:**
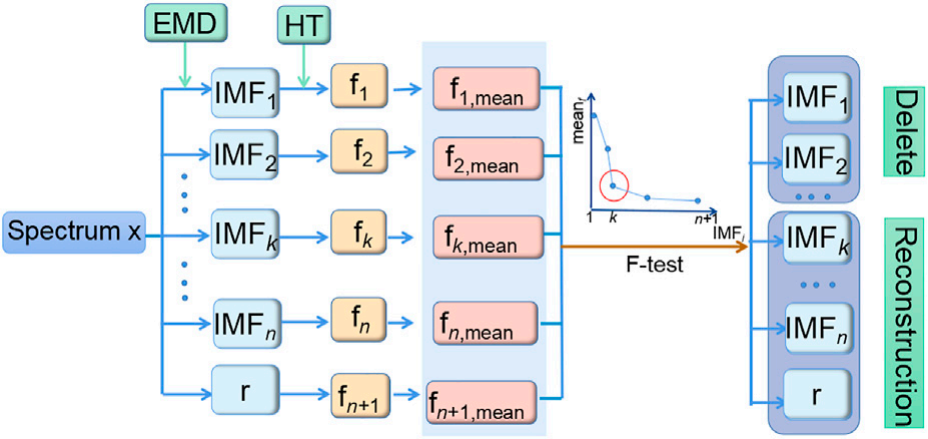
Flowchart of the spectral denoising method based on Hilbert–Huang transform combined with the F-test.

A novel method is proposed for spectral denoising based on EMD and HT. EMD is used to decompose the original spectrum into mono-component IMFs with different frequencies. HT is combined with F-test to determine the cut-off point of IMFs between noise components and non-noise ones. The denoising process of the method is shown in Figure 2. The corresponding MATLAB code and datasets can be downloaded from the website https://github.com/bianxihui/chemometrics-matlab-HHT-with-F-test. The details could be described as follows.

1) The original spectrum **x** is decomposed into a finite and often small number of frequency components containing *n* IMFs and a residue **r** by EMD. The low-order IMFs correspond to the high-frequency components and vice versa.
2) HT is applied to each IMF component and **r** to calculate their corresponding instantaneous frequency f_1_ f_2_ … f_*n*_ f_*n*+1_ by using Eqs 2 and 5, 6. Meanwhile, mean and standard deviations of f_1_ f_2_ … f_*n*_ f_*n*+1_ are obtained.
3) The cut-off point *k* is judged by Eq. 7,

$${\text{F}}_{k}=\frac{{\left({\text{SD}}_{k}\right)}^{2}}{{\left({\text{SD}}_{k+1}\right)}^{2}},$$ (7)

where SD_*k*_ and SD_*k*+1_ are the standard deviation of f_*n*+1_, f_*n*_ … f_2_ f_1_, and F_*k*_ is the ratio of (SD_*k*_)^2^ to (SD_*k*+1_)^2^. The significant difference of F_*k*_ is determined by the F-test with a 99.95% confidence interval. The degrees of freedom are set to 2 and 4, respectively. Furthermore, the F-test is being applied to distinguish a significant difference between the *k*th and (*k*+1)-th SD of **f**s. When F_*k*_ has a significant difference, *k* is judged as the cut-off point.

4) IMF_1_, IMF_2_, and IMF_*k*_ are the noising components that are deleted. The denoising spectrum is reconstructed by summing IMF_*k*+1_, IMF_*k*+2_, IMF_*n,*_ and **r**.

## Experimental

**FIGURE 3:**
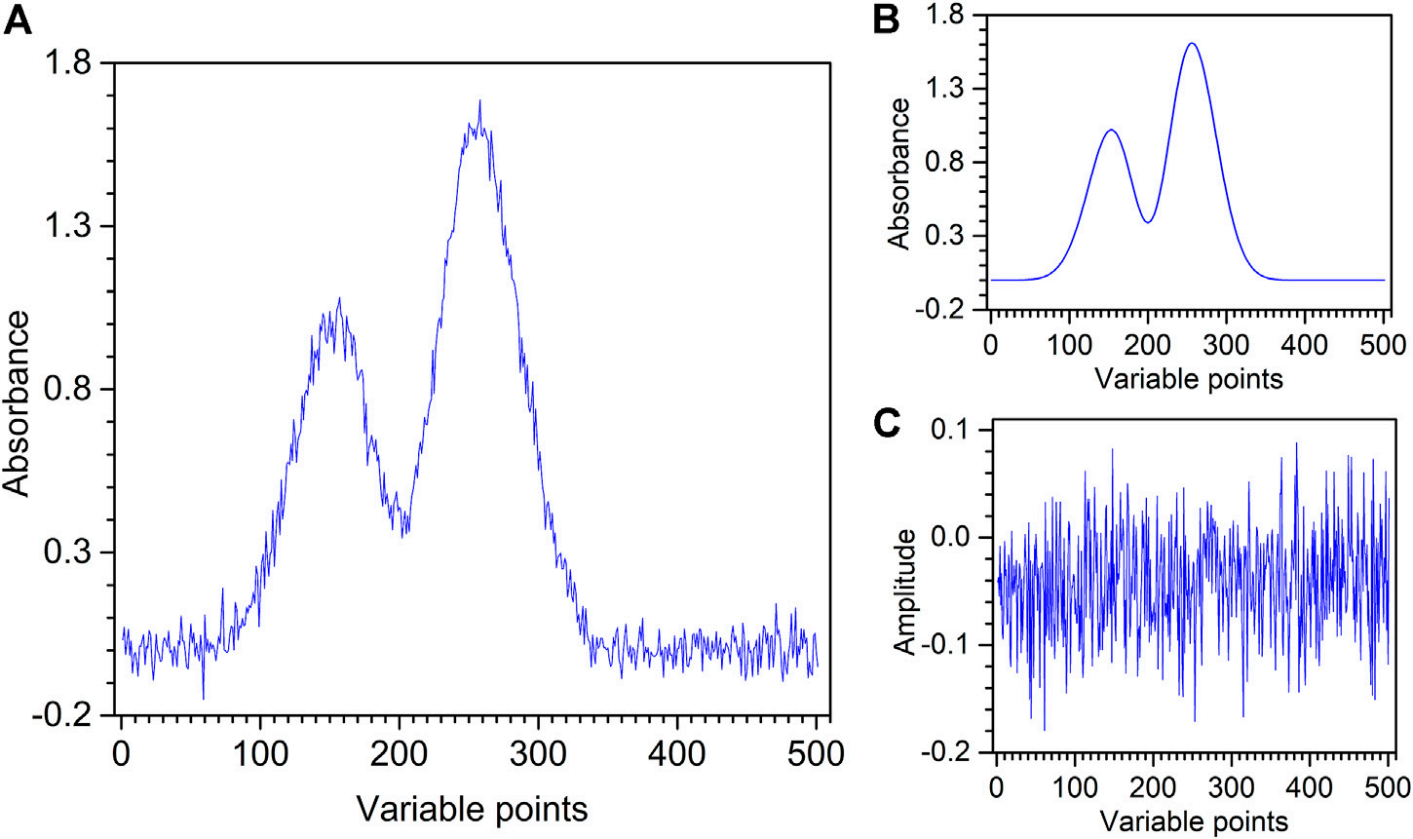
Artificially chemical noised signal **(A)** with its pure signal **(B)** and noise **(C)**.

The artificially chemical noised signal, XRD spectrum, and XPS spectrum are used to evaluate the performance of the proposed method. The artificially chemical noised signal is shown in Figure 3A, which is composed of 501 variables recorded in the range of 1–501, with a digitization interval of 1. It contains an artificially chemical signal with random noise in which the pure artificially chemical signal y_1_ is produced by the Gaussian function represented by Eq. 8, as shown in Figure 3B.

$${\text{y}}_{1}=2\text{exp}\left[-\left(\frac{\text{x}-2}{2}+4\right)\right]\left|\cos\text{x}-1.2\right|.$$ (8)

The noise signal y_2_ is added by Eq. 9.

$${\text{y}}_{2}=0.05\times \text{randn}\left(p\right),$$ (9)

where *p* represents the number of variables of an artificially chemical signal, that is, 501 values are generated. Moreover, randn generates values from a normal distribution with mean 1 and standard deviation 1, as shown in Figure 3C. The artificially chemical noised signal y = y_1_+y_2_.

The spectrum from the study by Wang *et al.* is measured on an X-ray diffractometer (D/MAX-RB, Japan) for catalyst materials (Fe/SCN) (Wang et al., 2021; Chu Y. Y et al., 2022). The diffraction angle range is 20–80°, the interval is 0.02°, and there are 3,001 variables.

The spectrum from the study by Chu *et al.* is measured on an X-ray photoelectron spectrometer (PHI 5700) for catalyst materials (FeNiOS-NS) (Chu Y. Y. et al., 2022). The binding energy is 700.08–740.08 eV, the interval is 0.1 eV, and there are 400 variables.

**FIGURE 4:**
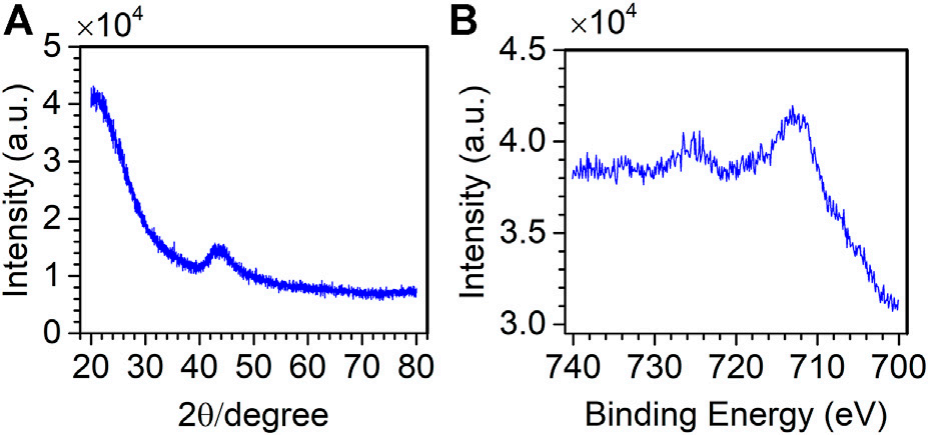
XRD spectrum of the Fe/SCN catalyst **(A)** and the XPS spectrum of the FeNiOS-NS catalyst **(B)**.

Figure 4 shows the original spectrum of XRD and XPS. By visual inspection, in Figure 4A, the spectral noise distribution is uniform, and the peak appears at the diffraction angle of 40–50 degrees. In Figure 4B, the spectrum contains sharp noise, which covers the peaks. Therefore, it is necessary to remove the useless noise and retain the useful peaks.

## Results and discussion

### Denoising of the artificially chemical noised signal

**FIGURE 5:**
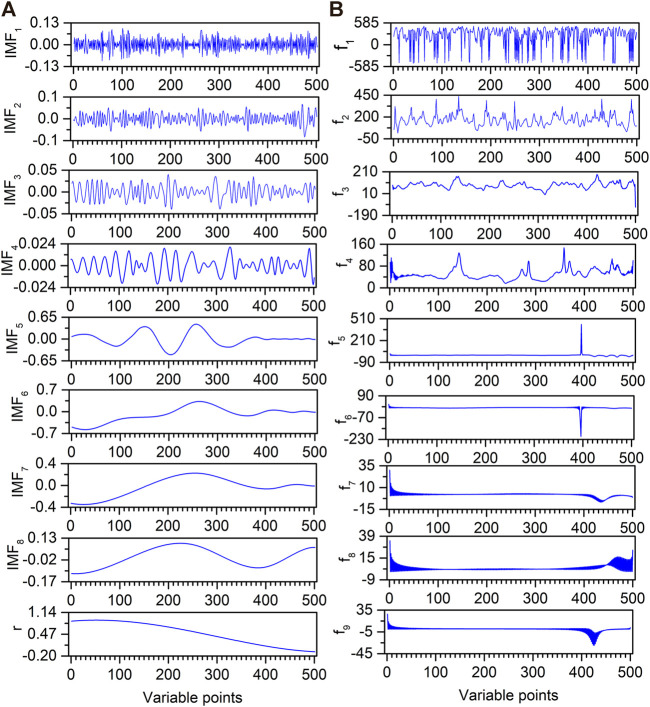
EMD decomposition results **(A)** and their corresponding instantaneous frequencies **(B)** for artificially chemical noised signal.

Based on the self-adaption and frequency decomposition superiorities, EMD is introduced to decompose the original artificially chemical noised signal. Figure 5A shows the decomposition result of EMD for an artificially chemical noised signal. The original spectrum is decomposed into eight IMFs (IMF_1_–IMF_8_) and an **r**. It is clear that the oscillation frequency decreases as the order of IMF becomes larger. By visual inspection, IMF_1_–IMF_4_ are obvious noise components with little information, while an **r** is the low-frequency components, which are extremely slow. However, it is difficult to determine whether IMF_5_ is a noise component or not.

**FIGURE 6:**
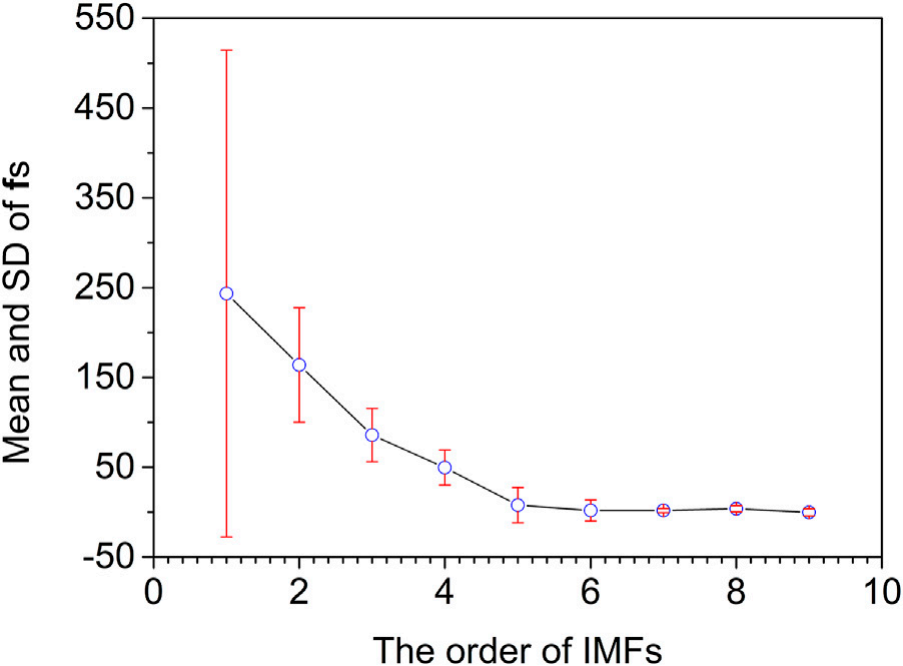
Mean and standard deviation of instantaneous frequencies for artificially chemical noised signal.

Different IMF components have different frequencies, and HT can be used to calculate their instantaneous frequencies. The HT results for IMFs of artificially chemical noised signals are shown in Figure 5B. The f_1_ contains a large number of peaks. With the increase of the f order, the number of peaks gradually decreases. Although the total variation range of the instantaneous frequency becomes smaller with the increase of the f order, the instantaneous frequency value for each variable is different for the same f order. Thus, the mean instantaneous frequencies of f_*n+*1_, f_*n*_, … f_1_ are calculated to observe the trends in frequencies. Meanwhile, the standard deviations of f_*n+*1_, f_*n*_, … f_1_ are further calculated for evaluating the noising degree of IMFs. As shown in Figure 6, the mean and standard deviation of f_1_–f_4_ are much higher than those of f_5_–f_9_. Furthermore, the mean of f_5_–f_9_ tends to be flat, and the standard deviation of f_5_–f_9_ is relatively low. Subsequently, IMF_4_ is judged as the cut-off point by the F-test. Therefore IMF_1_–IMF_4_ are deleted as noise, and IMF_5_–IMF_8_ and an **r** are reconstituted as useful signals.

**FIGURE 7:**
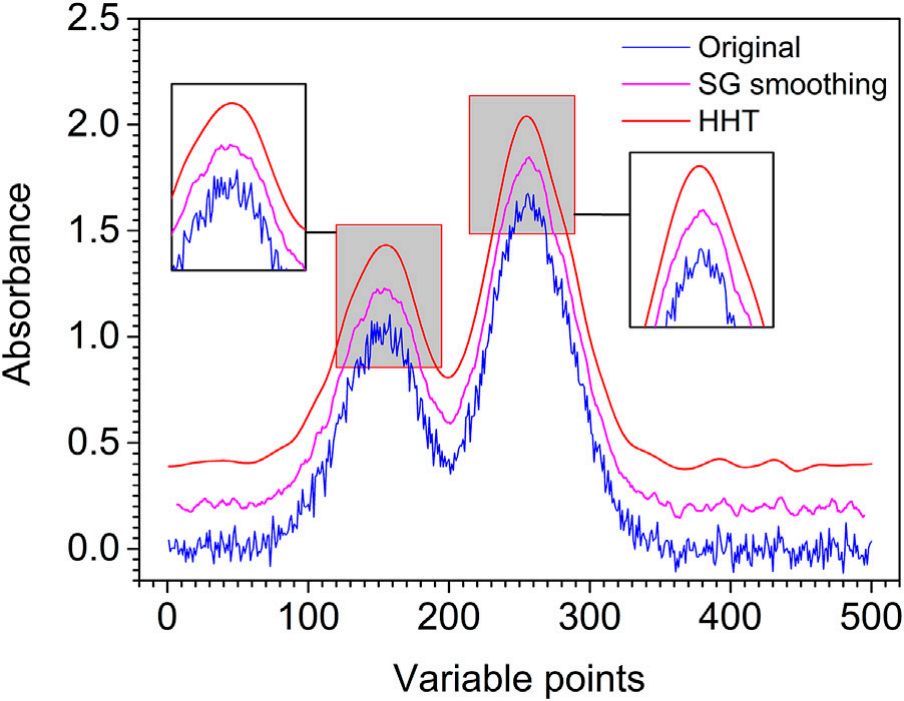
Original (blue line), SG smoothing (pink line), and HHT denoising (red line) artificially chemical noised signal.

To better evaluate the performance of the proposed method, SG smoothing applied to the artificially chemical noised signal denoising is compared with that of the proposed method, and SG smoothing window size is selected as 13. The comparison between the proposed method and the SG smoothing denoising results are shown in Figure 7. It is obvious from the figure that the spectrum is smoother and the peaks are more obvious after denoising by the proposed method. In order to illustrate quantitatively the superiority of the proposed method, the SNR of the two methods is calculated, where the SNR of the proposed method and SG smoothing is 22.59 and 21.60, respectively. It can be seen that compared with SG smoothing denoising, the SNR of the proposed method of denoising is improved and the denoising effect is more ideal.

### Denoising of the experimental spectrum

**FIGURE 8:**
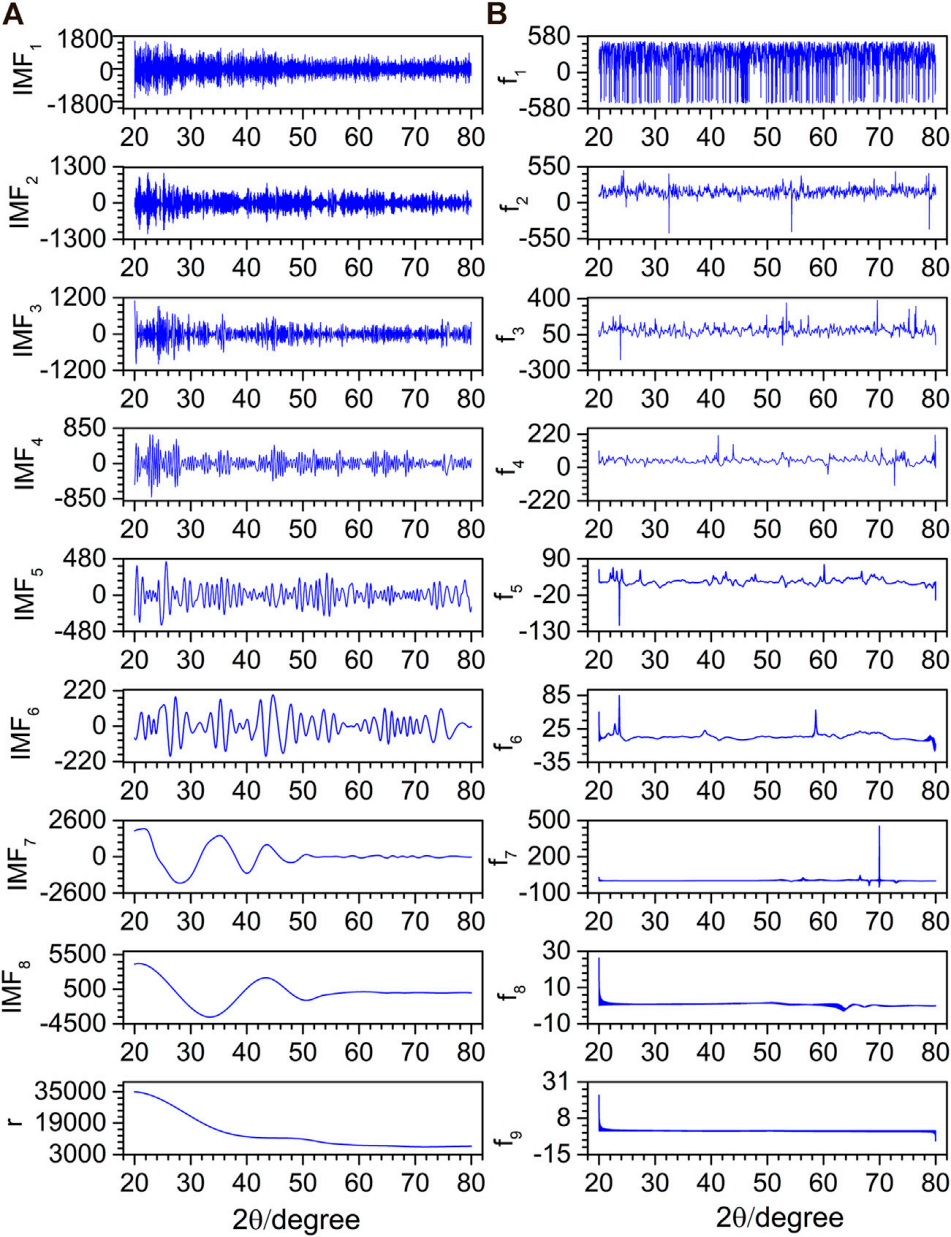
EMD decomposition results **(A)** and their corresponding instantaneous frequencies **(B)** for the XRD spectrum.

**FIGURE 9:**
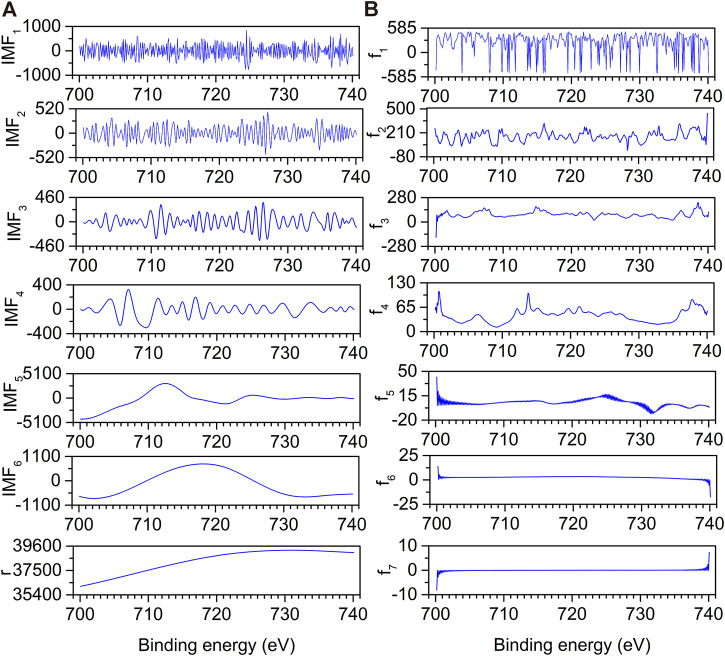
EMD decomposition results **(A)** and their corresponding instantaneous frequencies **(B)** for XPS spectrum.

To compare and verify the denoising effect of the proposed denoising method on the actual spectrum, noisy XRD and XPS spectra were selected for denoising. Figure 8A depicts the decomposition result of EMD for an XRD spectrum. The original spectrum is decomposed into eight IMFs (IMF_1_–IMF_8_) and an **r**. It is difficult to determine whether IMF_5_ and IMF_6_ are noise components or not by visual inspection. The instantaneous frequency of each IMF is obtained by HT, as shown in Figure 8B. With the increase of the f order, the number of peaks gradually decreases. Figure 9A depicts the EMD results of an XPS spectrum which is decomposed into six IMFs (IMF_1_–IMF_6_) and an **r**. The corresponding frequencies fs are calculated in Figure 9B. Similarly, whether IMF_4_ is a noise component or not, cannot be determined.

**FIGURE 10:**
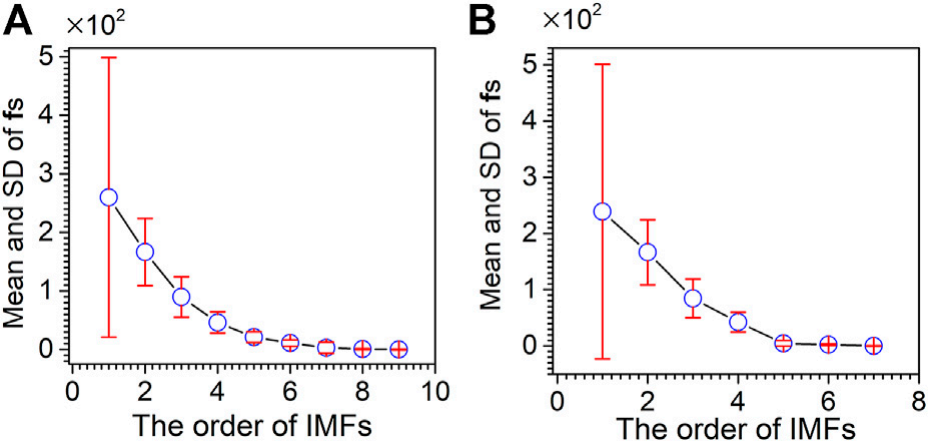
Mean and standard deviation of instantaneous frequencies for the XRD spectrum **(A)** and XPS spectrum **(B)**.

The mean and standard deviation of the f order for XRD and XPS spectra are calculated. As shown in Figure 10A, for the XRD spectrum, with the increase of IMF orders, the mean instantaneous frequency of f first decreases and then approaches being flat. The change in the standard deviation of f is directly proportional to the mean instantaneous frequency. The IMF with a large mean instantaneous frequency has a large standard deviation. Five is determined as the cut-off point by the F-test for the XRD spectrum. IMF_6_–IMF_8_ and an **r** are reconstructed as the denoising XRD spectrum. As shown in Figure 10B, for the XPS spectrum, the variation of the mean and standard deviation of fs is similar to that of the XRD spectrum. Four is determined as the cut-off point by the F-test for the XPS spectrum. IMF_5_, IMF_6,_ and an **r** are reconstructed as the denoising XPS spectra.

**FIGURE 11:**
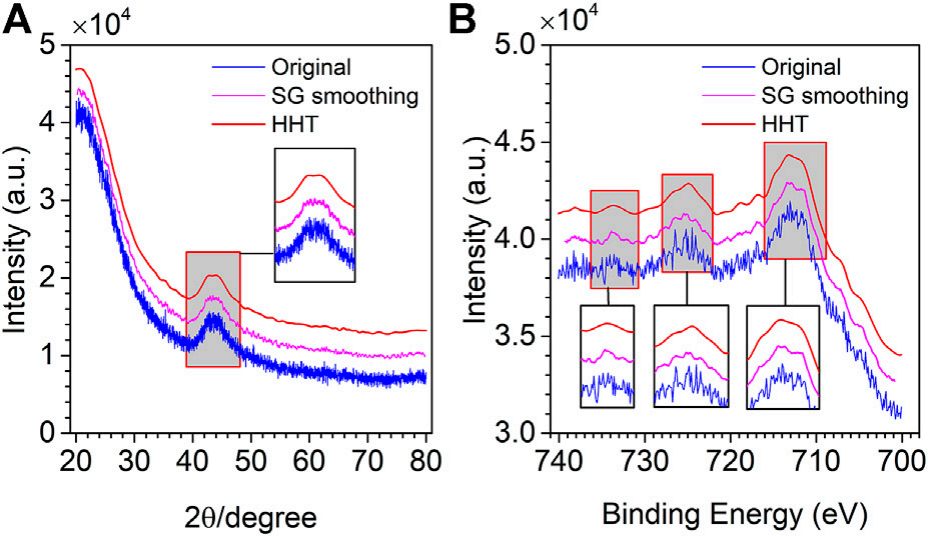
Original (blue line), SG smoothing (pink line) and HHT denoising (red line) XRD spectrum **(A)** and XPS spectrum **(B)**.

The SG smoothing is also applied to noisy XRD and XPS spectra. 21 and 17 are selected as the window size for the two spectra. Figures 11A,B show the denoising results of XRD and XPS spectra by SG smoothing and the proposed method. It is clear from Figure 11A that SG smoothing cannot remove the noise completely, and some noise is still left in the spectrum. In contrast to SG smoothing, the method proposed in this research not only makes the spectrum exceedingly smooth after denoising, but useful information is also retained. From Figure 11B, XPS spectral noise is also not completely removed by SG smoothing, especially at the three peaks. However, most of the noise is removed by the proposed method while retaining useful information.

## Conclusion

A novel denoising method is proposed based on HHT combined with the F-test. EMD is applied to adaptively decompose the spectrum without setting parameters. Then, HT is performed on IMFs to calculate the instantaneous frequencies. In addition, mean instantaneous frequencies are combined with the F-test as the criterion for distinguishing noise components and non-noise ones. It is concluded that the proposed denoising method is valid by noise removal for the artificially chemical noised signal, XRD, and XPS spectra. Moreover, compared with SG smoothing, the proposed method shows superiority both in observation and the SNR.

## Data Availability

The raw data supporting the conclusions of this article will be made available by the authors, without undue reservation.
